# Women, Administered Insecurity, and Multiple Alignments to Assuage Gender Inequalities in the Brazilian Zika Epidemic

**DOI:** 10.3389/fgwh.2021.716612

**Published:** 2021-12-03

**Authors:** Parry Scott

**Affiliations:** FAGES, Graduate Program in Anthropology, Federal University of Pernambuco, Recife, Brazil

**Keywords:** gender, Zika congenital syndrome, inequality, epidemic, alignments, administered insecurity

## Abstract

This article deals with the Brazilian Zika epidemic that started in 2015 and became an important event for understanding Gender and Power in health treatment contexts. It discusses a combination of reinforcement of gender normed care and therapeutic itineraries overburdening mothers and the construction of political awareness and practice associated with demands for state services. Working with a concept of administered insecurity elaborated to understand people displaced during the implementation of planned government development policies, it argues that the planned nature of health systems, despite their explicit emphasis on the objective of treating the health of the population, also generate administered insecurity as planned administrators of scarcity. It uses data from a 4-year research project entitled “Doing Ethnography on Care” in Recife, Brazil, to show the multiplicity of contexts that are brought out through the practice of mothers in providing care for their Zika-syndrome stricken children, and how they reflect varying power relations that constantly re-dimension maternity along lines of gender relations in different institutional domains of treatment and research. The severe gender inequality in caring for infants was partially assuaged by multiple alignments made by the mothers and infants promoting dialogue and practice in varied contexts, including interaction with the research team. Family and Community Relations, Mediation, Favors, Accusations, Judicialization, Collective Action, formation of Associations, and learning to produce videos are seen as constructors of complex meaning and practice of mothering in a way in which gender goes beyond the limits of an overburdening practice of care. Gender provides a significant difference for mothers whose knowledge and familiarity of domains of health and health-related provision of services and knowledge, forged in their search for treatment for their child, create and legitimate spaces for the exercise of informed citizenship and a sharp awareness and resistance to practices by the state and other agents who administer insecurity. The final remarks synthesize some of the more important reconfigurations of gender relations and power in the context of the Zika epidemic and alerts to the challenge of the transitory nature of an epidemic and the gradual, and not so gradual, exhaustion of institutional interest for the dilemma of the mothers and also shows the role of anthropological research in promoting gender equality in epidemic contexts.

## Introduction

The Zika epidemic raises questions of how to reinforce gender and the relation of research practice to diminishing gender inequality. From 2015 to 2017, the Zika epidemic became a health issue of national and international concern, severely affecting mothers and children. Our research team in the state of Pernambuco accompanied daily activities and therapy itineraries for over 5 years. The practical results of the research were to document the process and to promote a fruitful and respectful dialogue between mothers and health service professionals to value how mothers see and deal with this health problem. What might be gained by them despite the evident gender inequality stemming from the heavy load of care?

The first section discusses limitations, possibilities, and processes of the empowerment of women to underpin a usage of the ideas of multiple alignments to face administered insecurity and value actions far beyond daily care along therapeutic itineraries. The second presents the research methods and describes the population. In the next session, these ideas guide a description of the varied scenarios confronted by mothers in six different contexts encountered along their itineraries. The final section deals with the articulation between the practices of women and anthropological research in understanding how to combat gender inequality in such epidemic conditions.

The Brazilian Zika Congenital Syndrome (ZCS), identified in 2015 by medical professionals in the Northeast of the country and rapidly well publicized in a book by Diniz (1), is a severely gender-marked epidemic. Zika itself, when contracted by men, women, and children, is easy to pass unnoticed. It is an Arbovirus whose vector is the *Aedes aegypti* mosquito which proliferates in many unsanitary contexts, especially in tropical climates^1^. The symptoms are a short-lived feverish feeling over a couple of days or more and a skin rash that also recedes after a few days. Two other diseases that bring on great discomfort and can lead to death, dengue, and chikungunya are also transmitted by the same vector. The discovery of the comparatively “mild” Zika as having serious consequences only occurred when an unprecedented high number of babies with microcephaly were born in the last months of 2015. It was so alarming that on November 11, Brazilian authorities declared a national public health emergency, and the WHO extended it to an international scope in February of 2016.

Zika infections are more reported for women than men not only because of epidemic periods but also because of sexual transmission being underreported since diagnosis occurs at birth or somewhere in the reproductive cycle of women (2, 3)^2^. This is one of the factors that hides male-originated Zika infections that produce ZCS. Male and female babies were equally likely to present not only microcephaly, but also some of the strew of other consequences that made up an individually variable in addition to a collectively repetitive set of impediments to childhood health and development: irritation and hyper-excitability, neurological abnormalities, gastric and deglutition disturbances, convulsions, hearing and vision limitations, hypertonia, and ambulatory difficulties, among other problems [see (4)]. All of these problems became visible around birth and infancy and implied a prospect of a future of continued severe limitations and intense dependence on offspring. What was at stake was the very gender-marked capacity of each woman to deal with the new situation in which her child redefined her priorities and almost always determined a new life course. The fact that she gave birth led to questions about when she had the rash characteristic of Zika infection, but almost never to the question of whether her male partner had presented a rash. Nothing can hide the intensive gender inequality that relegates women to a “sacrosanct” role of a loving caregiver. Given this reality, measures to combat gender inequality inevitably take into account this situation of super-valuing of motherhood and care for a newborn infant with ZCS. The presumption is that these women, deemed as responsible for infecting their babies, will have to care for their children, even if it means full-day daily commitment, loss of income from work, reorganization of living arrangements and conjugal relations, among other activities that may best be seen as “sacrificial” and “unequal.”

Two questions are addressed in this article. The first is, in a context of an event that severely heightens gender inequality, how is it possible for women to find ways to seek reinforcement for themselves? The second is, how might research practice contribute to this reinforcement? The women studied are mothers overburdened with the responsibilities of caregiving as a consequence of their children being born with ZCS in Brazil.

## Caregiving, Multiple Alignments, Administered Insecurity, and Therapeutic Itineraries

These research practices have to do with our understanding of gender equality, multiple alignments and insecurities, and inequalities created in daily perambulation along therapeutic itineraries. Mothers, families, and health-related professionals act in contexts permeated by hierarchies of care with each one presenting specific characteristics.

Three perspectives in our approach are highlighted: multiple alignments, administered insecurity, and therapeutic itineraries. All are discussed in relation to the unequal gender relations of mothers devoted to caregiving.

The first, mentioned above, is that one manner of effectively combatting gender inequality is by way of practices that are a) collective and b) multi-facetted. Both Cornwall (5) and Sardenberg (6) point out that the idea of empowerment as a concept emphasizes the means of producing women who become more capable of making their own decisions and acting in favor of themselves and of women in general in private and public spaces. Cornwall alerts to the degree to which the concept has been appropriated by the individualistic and entrepreneurial aspects of social involvement, dulling the perception of differences based on gender as the content of power structures that need to be questioned. Batliwala (7) makes this clearer, emphasizing that empowerment requires a recognition of the surrounding world and the position and condition of the agents in it from their point of view, yet aware of the need for changes. That is combined with an effort to create power “with” other women, and not simply individual gains. We see these practices in the *multiple alignments* that are produced as they search to cope with being a mother of a severely dependent child. These ideas are applicable to an experience-grounded and contextual approach of viewing what diminishes gender inequality because neither alignments nor contexts are fully predetermined.

This experience is informed by an anthropological, methodological, and ontological practice of co-involvement with those with whom we are interacting. During the process of choosing the research preferred contexts of observation, it was found that they may always be complemented by additional unforeseen contexts that arise in co-experience in a way in which we extend the understanding, range, and reach of what we seek to understand. This procedure unleashes researchers from their dependence on the isolation of specific questions to be researched and dealt with in the field. The discovery of new ways of dealing with varying social contexts^3^ that are present in daily living helps find multiple alignments and shifting narratives with the maintenance of core solidarity with those with whom we do research and share many convictions about focused issues. In that way, from the beginning, researchers may find ways to contribute to a diminished collective inequality. Said in more direct terms, the more ways in which anyone can interact with different groups that in some way develop practices which share different, relatively coherent, notions of diminishing gender inequality the more likely that things will “work.” Multiple alignments enlarge the scope of possibilities of advances. This is what is called multiple alignments in this article. It is a way to create power “with” but does not circumscribe the relations to any single sphere of cooperating agents. It is another way of seeing how intersectionality, described by Crenshaw (9) and Collins (10) who emphasize multiple sources of discrimination, also becomes a tool for collective action.

Another standpoint throughout this article is that activities between providers of services and families are interactions permeated by administered insecurity. Those who are more powerful in any specific hierarchical context make part of what Vianna and Lowenkron (11), inspired in Das and Poole (12), identify as the dual production of gender and the State. State-making and order-making occur through the many ways in which people interact with organizations and individuals creating politically marked citizens who both search for support and resources and find themselves to be dealt with in accordance with hierarchically ordered categories of belonging. From this point of view, administrative practices, however, strewn with references and intentions of collaborative and strengthening measures, contain bureaucratically embedded humiliations and a generation of insecurity that preserve and construct such hierarchies. This is an elaboration of the characterization of Herzfeld that institutions in bureaucracies generate indifference and, also, reinforce inequalities. In that way, using the terms of this article, the two elements – mutual alignments and administered insecurity - coexist in specific ways in each context. The idea of administered insecurity was developed in previous ethnographic studies on development projects that almost always resulted in the dislocation of the population, with an emphasis on more losses than gains for local populations (13). In these cases, when the State, or other promoters of projects in name of the State or a notion of development, get near the population, there is an emphasis on the expectation of losses for the local population, even when such losses co-exist with gains.

A policy of public health (or education, or welfare), which provides services for the population, does not immediately generate the same kind of insecurity as development or urban renewal projects.^4^ As a policy, there are concrete services offered locally, in name of the needs of the population and not of some commodity or resource being circulated. However, it is important to point out that such services related to policies do not come simply as a redistributive gift from the State.^5^ As many researchers and social movements have pointed out, gender and state have a dual production, or as Vianna and Lowenkron (11) put it, the State is “a web of meanings, possibilities for action and forms of interdiction made from and by gender dynamics.” Developed in this same perspective from both sides of this dual production, Freire (18) examines the precariousness of the services and the demands of the health system and emphasizes the need to process juridically the State in many cases. Fernandes (19) shows how codes and meanings in daily gender relations perpetuate inequalities that perpetuate freedom of movement for males and restrictive care activities for women, constantly reinforced as men and women deal with the State.

The common grounds of submission to indifferent bureaucracies (20) contribute to similar feelings of indignation and humiliation, which are other ways of producing institutional violence and “administering insecurity.” They become omnipresent in face of a state apparatus that reflects scarcity and selective treatment as it works on the regulation of both itself and the population. The range of ways of conveying feelings of disrespect, inadequacy, debt, and outright violence becomes wider and more evident as the multiple contexts of interaction, in search of resources and support, sum up to a repeated devaluation and regulation of the person, very much in function of markers of social class, ethnicity, residence, race, and, of course, gender^6^.

The idea of therapeutic itineraries, when seen from the perspective of service users, does not imply an ordered following of directions of health providers. Much to the contrary, it offers many different arrays of relations that are found as valuable to deal with their search for resources and support, each in their way. Families, neighbors, religious groups, associations, clinics, hospitals, social security offices, transportation services, routes, etc., all are places on the itineraries in the case we are examining. Each contributes differently to the ordering of a multifaceted identity as a caregiving mother (22) Bonet (23) prefers the idea of itinerance to emphasize both the prevailing precariousness and the different routes that may be preferentially followed by subjects in search of resources and support. Itineraries are not blueprints of how to act; they are lived experiences in different contexts.

It is very common to have individuals who develop their activities under the auspices of the State, private and philanthropic institutions, and non-governmental organizations, but who mediate relations with citizens in a manner of which these citizens understand as being favorable for their demands. In those same contexts, in which the administered insecurity is constantly reaffirmed, the formation of multiple alignments with sensitive professionals (who also may be researchers) undo some of the deleterious effects of insensitive actors and structures. Often, these “mediators” are capable of sharing their specific insertion in more than one bureaucratic power network in a way that weakens the gender (or other) inequalities inherent, institutionally, in them.

Before going to a sequence of narratives that show how multiple alignments worked to diminish gender inequality in contexts of administered insecurity in providing services for victims of ZCS, a few words must be dedicated to some of the consequences of these relations being developed under the extraordinary conditions of an epidemic. An epidemic creates highly visible and explicit priorities that intensify external surveillance of the functioning of institutional organizations and local capacities in responding to the threat of a potential expansion of health problems to powerful areas otherwise less affected and, above all, extremely interested in keeping the problem contained and at a distance. Funding abounds in the form of cooperation, collaboration, investments, and grants, accompanied by sharing of personnel and technical expertise, steeped in the moralism of humanitarian discourse (24). Such external participation creates an aura of accountability which spotlights everything related to the response to the epidemic. Local and national identities are at stake depending on the performance of research professionals and health and service bureaucracies seen through the lens of the health of the population and the controllability of the problem. Depending on the evaluation of responses as positive or negative, the epidemic can reinforce or weaken the image of diverse sectors of the local population and administrative bureaucracies articulated in larger networks, along with becoming a factor affecting the relations of countries in the arena of international relations.

In the case of Zika in Brazil, the rapid recognition of the problem and scientifically competent response quickly mapping the characteristics of the syndrome, together with a rapid fall in its occurrence along 2016 - the first full year of addressing the problem - led to the lifting of the international epidemic alert in November 2016 and National alert in May 2017, and a recognition of the quality of many agents in the Brazilian response (25). Efforts to treat the problems of the affected population were multiple and defined priorities that enabled many to find therapeutic treatment. At the same time, in general, such services were subject to a more questionable judgment about the nature of the institutional response. The most questioned were access and quality of care, scarcity of material and equipment, and other technical and professional services. In Pernambuco, by 2018, over 450 mothers and women caregivers found themselves enmeshed in a life-long, not-so-quickly solved situation of super-dedication to the care of a handicapped child with severely limited chances of ever becoming independent of the need for care (26). They, certainly, were stricken by a reinforced overburdening of gender inequality of a long-term nature. The severity and degree of suffering do not become assuaged over time, but accompanying these women over 4 years, it was clear that in many ways they had gained a diminished inequality as active women in many aspects of their lives. This had to do with the multiple alignments (including those with our researchers) that they, and those who were in touch with them, made to respond to administered insecurity as they sought to strengthen their domains of knowledge and experience in response to the epidemic.

## The ZIKA Epidemic And Research Methods

From October 2016 up to the time of this writing (April 2021), our research, called “Doing Ethnography on Care,”^7^ has been in contact with over half of the more than 450 mothers of children with ZCS in association activities, health treatment institutions, and occasional other events. We worked closely with a more reduced group of more than three dozen women. We established greater affinities, usually through associations and, at times, introduced by other mothers or met during their treatment and therapies. We accompanied their therapeutic itineraries and daily social and spatial mobility. Over 45% (26) have a family income below one-fourth of a minimum wage per capita in the household in the unified state registery (CadUnc). Almost all belong to one of the associations, some very active in varied fronts, others only because of the occasional donations that are distributed. Some of the mothers, harder to quantify (perhaps under 10 %), decidedly avoid all this contact, be it with associations or even with health institutions. We concentrated our fieldwork in the Recife Metropolitan Area but made occasional trips to more distant municipalities. After knowing the women well, we recorded interviews with over 40 mothers.

Besides interviewing managers and policymakers, we used observation (principally while accompanying mothers) and interviews to understand governmental and non-governmental services of health, along with, on a lesser scale, mobility, education, and social welfare. These totaled more than 50 interviews.

We registered observations in field notes and discussed them in meetings of the research team. The recorded interviews with the mothers that became our friends and with service professionals, administrators, and researchers all proved very valuable (27, 28). Rather than a fully protagonistic search for the empowerment of women in the name of combatting gender inequality, our approach was to discover the social relations that formed around their daily practice as ZCS mothers and try to catalyze, in both their and our view, what was most beneficial to them, aiding their viewpoints to become better known by all. That approach also led to our promotion of specific activities of video production, discussed in this article from the point of view of its contribution to reducing gender inequality.

With a team of five senior researchers, six graduate students, and six undergraduates^8^, we understood one of our key objectives to be finding or creating spaces for mutually respectful dialogues between mothers and their families, on the one hand, and administrators and professional practitioners and workers, on the other. Our expectation was that our presence as a group of researchers could contribute to associating a deep knowledge of the experience of women and their family networks affected by the virus to an effort to widen sensitivity and response of the health system in a way in which it incorporates mutual knowledge and collaborative practices (*Doing Ethnography on Care* research project).

Such expectations are intersectional because they involve gender, race, class, and bureaucratic structures in the multiple spaces that could be observed at many levels by so many researchers and be understood as making up part of a constant reconfiguration of the practice of citizenship for these mothers. As the research unfolded, the large community of researchers from different fields attracted to the epidemic situation became another important group about and with whom we do research.

## Gendering Experiences of Multiple Alignments and Administrative Insecurity: Observations From Fieldwork

Our ethnographic data shows the multiplicity of therapeutic and everyday life contexts brought out through the practice of mothers in providing care and resources for their Zika syndrome-affected children. They reflect varying power relations that constantly redimension maternity along lines of gender relations in different institutional contexts of treatment, research, and daily living, as has been examined elsewhere in our research (22). From the beginning of the research, the overwhelming burden and severe gender inequality in caring for infants for the mothers of Zika-affected children were present and it never receded. But when the writing, observation, and discussion about what we were seeing and sharing with the mothers is closely examined, it also became clear that such inequalities were partially assuaged during the daily practice of co-participation in multiple alignments with the mothers and infants as they followed their strategies of multiple therapeutic itineraries, made even more visible since they are significant results of our research perspective. Dialogues, negotiations, mobilizations, priority treatment, and favors proliferating in many different contexts created a favorable field of individual and collective agency which, more often than not, contributed to a disposition to act. In general, this contributed to and was reinforced by, the formation of an intense relational network with other mothers with parallel experience in associations that played a leading role in finding responses to their needs in a way in which they felt strengthened as women.

A short array of these intertwined contexts which were relevant for our research questions, emphasizing observations and narratives of experience, are presented here to show transformations in gender inequality of repeated and differentiated sets of practices of these mothers. Each one of these contexts of practice deserves a much longer ethnographic description and discussion than can be given here. Even when inflicting the suffering characteristic of administered insecurity, it is possible to see how shifting multiple alignments permitted different degrees of diminished gender inequality in each set.

### An Opening Seminar

Local research colleagues with a grant from a prestigious British funder of health research invited our recently formed research team to their (and our) first seminar concerning the social impacts of Zika (slightly more than a year after the declaration of the national emergency). The Project was coordinated by women researchers working with others at the well-articulated Oswaldo Cruz Foundation Recife campus^9^ as participants in a multi-professional “Microcephaly Emergency Research Group- MERG.” This group was quickly formed at the beginning of the new epidemic and capable of capturing financing and exchanging important research findings, especially to deal with the epidemic. In the 2 days of the event, besides internal articulation with their British partners, they produced talks with technical information about the epidemic itself. They also invited representatives of two associations that represented the Zika mothers. Additionally, since they were feminist activists, they included a special discussion with a spearhead group of five local social organizations (NGOs and Community organizations) that had gotten seed funding from *ONU Mulheres*, the United Nations Women's Organization, at the outbreak of the epidemic to think about feminist ways of dealing with it. Each of the groups showed how they dealt with disseminating information about Zika combined, invariably, with information about contraceptive practices and reproductive health. A clash of perspectives rounded the meeting because while one Zika mothers' association (AMAR) cautiously and firmly advocated the need for “caring for caregivers” and development of home-based craft skills for the mothers (omitting questions of reproductive health), the other (UMA) overtly emphasized that, together with caring for the needs of the mothers and children, they were firmly opposed to abortion as a right of choice for women. Without going into details, what was evident was that taking a pro-right to abortion stance would be a double affront to the mothers. First, it would question why they did not choose to abort, and second, it would suggest that their live child could easily be seen as a source of discontent.

The historical path of continuing to follow the very important emphasis on women gaining control over their reproduction rights and practices (29) to reduce gender inequality would have little immediate reverberation for these women whose situation was to understand how to deal with the new life course of extended dedication to caring. Hearing an indirect and unnecessary critique of their choice (when the choice was available^10^) makes this part of the feminist narrative take the form of administered insecurity, eroding the value that they are giving to care and to love of their children as positive traits during the process of inclusion in a research effort. That is not to deny that the discussion may open new perspectives where changes in sexual and family life certainly occurred and in which there is evidence both of controlled avoidance of pregnancy during the epidemic period, and of differing post-epidemic decisions to avoid more children or to promote a new pregnancy.

A clash about what parts of the feminist agenda contribute, in fact, to an effort to diminish gender inequality does not invalidate feminism for these women who are befriended by researchers and professionals and are able to show the specificity of their demands for more access to better services, valuing of care (even though it burdens them), and finding ways to care for caregivers. The seminar made their demands known in a participative debate in a university setting and seeing the representative leaders of their associations as highly legitimate participants in the discussion is a form of diminishing the sense of silence and invisibility of the dilemmas they face. Those who are aligned with them discuss reproductive and sexual rights, but opportunely leave aside the open discussion of these rights when the subject tends to imply a negative judgment about their decisions.

### Mediating Limits of Institutional Priority: Favors and Collective Action

Because of the epidemic complex, mothers of children with ZCS are not just simple patients or users of public services. They have explicit, written priorities in several areas elaborated by authorities who use their understanding of the situation and capacity to dialogue with families and their representatives as a clear emphasis of the collective response of public services, giving priorities to these women across their experience in therapeutic itineraries. They may mark exams and medical appointments and do their consultations before other patients who are awaiting their turns. Their inclusion in registers for income benefits also is given priority. Specifically, they have guarantees of the concession of apartments in housing projects. They are also invited to complex multiple intervention sessions (called *mutirões*) with many laboratory exams and individual and collective therapies and consultations promoted by researchers and therapeutic institutions. Their new generation wheelchairs or orthopedic apparatuses are made more easily available as donations. Alternative transportation for handicapped populations is furnished more rapidly through public transportation programs and their children are to be given preference in programs of inclusion in day-care centers.

All of these priorities are accompanied by contradictory measures and means that make the prioritized families, grateful for the importance given to them, also feel uneasy. Normally they are the fruit of a combination of scarce resources and of embedded institutional and individual hierarchizing between those who serve and those who are being served. Long waiting times, precarious systems of communication, difficult availability of documents proving need about low income and diagnostics, omission in return of results of exams for their private information (generating feelings of being treated more as test animals rather than human patients with needs^11^), severe restrictions on usage of special public transportation and questionable quality, the negation of presence of helpers in many situations, lack of personnel or equipment to offer needed services, limited access to donations of medicine, hygienic material, food, etc. What is lived daily is an insecure priority and a constant reiteration of the subordination of the women and their children to a relatively insensitive bureaucratic network.

Returning to the argument of Vianna and Lowenkron about the double production of the state and gender (2018) reinforced by other intersected categories, the State is capable of touting how it has given priority to these women, at the same time that access to these priorities, in practice, comes framed in disrespect, scarcity, and the continued application of restrictive regulations and codes. At the same time that priority is given, subordination is reinforced.

There are several ways that multiple alignments have contributed to diminishing the harm inflicted by this array of restrictions which lead to a very diminished idea of “priority.” One resorts to the use of favors in which personal networks of sensitive professionals (in this case, almost all women) produce a mediation in which subjects' collective priority is put to a more individualized use, particularly, building more extended distinction for these individual mediators in their professional practice and putting into effect what should be an institutionalized priority and not a favor. Another is taking the question to the associations that represent Zika mothers and children and using their communication networks to collectivize the demands. Researchers become fully involved in both these processes, helping to make telephone connections, send WhatsApp messages, give car rides to avoid the difficulties of public transportation or pay for such services, and, in general, help systematize demands to make them more visible, coherent, and collective. When the practice of promoting obedience of announced priorities seems more practice of conveying marks of subordination and devaluation on the Zika families, once again the forming of multiple alignments, formed above all among women professionals and mothers, becomes a feasible response to reinstate some positive aspects of priority and solidarity among women.

### Mobility and Social Networks

Institutional response to health and educational needs is centralized as part of an administrative expectation of rationalizing the delivery of services, especially in state capitals, as is the case with Recife. But families stricken by Zika come mostly from poor urban neighborhoods and, also, from communities distant from the state capital. Barbosa et al. (26) demonstrate the overwhelming poverty of most Zika syndrome mothers, calculating that 90% of them are included in the Welfare Registry^12^ and half of them with proven low enough income (1/4 minimum wage per capita) to qualify for a Continuous Payment Benefit (BPC).

Forty-nine percent reside in the Recife metropolitan area where health and social services are concentrated, and almost one-fifth reside in rural areas. For those who live more than 150 km from the metropolitan area, travel to the city for health care is offered by the municipalities in busses and vans, but for those who live close enough to take advantage of this, such travel becomes physically exhausting because of the long hours (i.e., starting early in the morning, stopping on the route in institutions where others will be treated, getting to therapy well before the time of treatment, waiting for treatment and the arrival of the vehicle for the return trip, and then traveling back and arriving at night). Some women endure for many months as many as three or more times a week (generated by a feeling of insecurity since if they miss three sessions, they know they will be cut from the list by the institution). Some abandon the treatment and others decide to move to the city. But in all cases, the social network of the mothers, (family, relatives, neighbors, friends) is severely affected by having to submit to a routine of multiple therapies and consultations in the city, and almost always in different institutions because of the specialization (i.e., physical therapy, occupational therapy, gastroenterology, ophthalmology, pediatric neurology, etc.) (32).

These women find that a great number of those who are closely related to them do not seem to know how to deal with the limitations of their children, particularly treating them either with excessive carefulness or with outright discriminatory comments that demonize their children. The result is a retraction from interaction with the overly careful or overtly accusatory persons in their networks, and almost always a very strong, selective, alignment with mothers, aunts, sisters, and elder daughters whose qualities of solidarity make them be hailed as extraordinarily solidary supporters. To these limited in number but very close supporters, other mothers are befriended in therapy and consultation sessions and, above all, in associations dedicated to Zika children and their mothers. The force of these strong alignments among women, who are willing to participate in the collectivization of the demands for care and emotional bonds, reduces the isolation that might otherwise be installed in their daily lives at the same time that it leads to emotionally costly exclusions.

Among those who are most prone to become absent are husbands and fathers who have difficulties in dealing with demands for childcare and with a likely reduction of the marital relationship and, above all, their freedom to circulate [reinforcing the gender code of under-involvement of men in care activities – (19)]. Many women put much emphasis on this loss of male support in a way that has two different outcomes. One is the strong emphasis on being or becoming a female single parent, home dependent on intense interaction with other women (one of the associations repeats constantly that 79% of their women members are household heads that cannot count on the participation of men). The other outcome is, for those who continue with a participating spouse, the recognition and praise of what tends to be lesser active participation than that given by other women (33). In that way, some of the few men who stay with their partners gain recognition for their dedication in many cases well beyond the dimensions that such dedication results in allaying the burdens of mothers for the care of a “special” child. Male absence, be it physical or attitudinal, relegates care almost exclusively to women, reinforces patriarchal gender inequalities.

### Associations

The complicated and often very expensive access to exams and treatment that demand specialized knowledge, technology, and facilities for children and adults that suffer from rare diseases led an upper-middle-class mother with a child with a rare disease to form an association called AMAR (“Love” is the acronym) in 2013 to bring together a variety of families that care for family members with rare diseases and that support their many demands. This de-individualization of demands expands the understanding of pathologies and support for citizens and forms new networks of cooperation and visibility. It promotes a concentration of means of access to complementary and multi-sectorial services (not only in the fields of health, but of law, education, pedagogy, jobs and skills, recreation, transportation, philanthropy, and social services), and, above all, provides a circle of caregiver colleagues (principally mothers) with whom to interact as was mentioned in the previous item.

After 3 years, early in 2016, the new “rare” disease of Microcephaly caused by the Zika virus brought into the association a numerous population of “micro” babies [refer to (34), Fleischer and Lima (35)], centering the attention on them. At the same time that it brought public recognition of the demands of the AMAR association, it brought a distortion that overemphasized the plight of those stricken by the effects of the new epidemic for which resources and efforts were disproportionally more available. A mother with a “micro” child and participant in AMAR formed a new association called Union of Mothers of Angels (UMA) which worked more specifically with families affected by the new pathology and was able to centralize many of the relatively abundant (yet still insufficient) services and form an ample network around them.

Most mothers who were involved in one or the other (and sometimes both) of the associations were effusively enthusiastic about the solidarity received from being together with other mothers who lived a similar experience and therefore had a better understanding of the situation. These groups were the path to a new alignment, of having “power with,” that counterbalanced their own diminished social networks, serving as a preferred space for sociality and access to resources that brought on a transformation of the condition of individual victims, into a collective citizenry reinforced by the multiplicity of areas of their demands. More than other women from popular neighborhoods, their lives were constructed by their relations to state and service bureaucracies and, inversely, they became key figures in the forming and reconfiguration of these services and the operation of networks of mediation that put them in touch with one another for the common goal of reinforcing the care domains^13^ of each other. These mothers, aligned with the associations, boosted by the epidemic situation, are no longer simply individual patients, but rather become key figures for the creation of a reputation of competent professionalism and the creation of evidence of successful systemic response of services and knowledge for the state and the country. New paths for public visibility and consciousness of citizenship appear. As members of families stricken by the effects of a specific illness, their citizenship is marked by a dependence on special efforts that occurs with other bio-identity associations (of children with cancer, of leukemia, of sickle-celled anemia, of patients who had the transplant, etc.). One must have an extraordinary source of individual and group insecurity in order to become involved in these networks of multiply-aligned actors that are concerned with contributing to the administration of their well-being. This occurs within the limits of the demonstration of continued sacrifice or continued insecurity.

### Processing Legally for Welfare Rights

As is elegantly argued by Freire (18) and by many other observers of health and welfare service and confrontation of gender violence (37), the extraordinary daily scarcity that characterizes what ideally would be a universal system of access to health in Brazil (SUS – Unified System of Health) and to deal with gender violence generates a repeated practice of processing the state juridically in order to get access to resources that are little more than the rights of those seeking care for themselves and compensation for their suffering. Proving that the state is at fault for the spread of pathologies or is negligent in its understanding of welfare needs stemming from those pathologies, it may be possible to have demands recognized, and that is what happened with families affected by the ZCS.

The multiple alignments of mothers of children with Zika kept them in direct or indirect contact with mediating professionals, researchers, journalists, other mothers in the same situation, association administrators, and politicians and administrators in a parliamentary front to deal with a deficiency that brought questions to the state legislative assembly and attending commemorative events. Caring for their children was far from being a private concern. Eventually, it became a public issue. The epidemic context contributed to the practice of sharing the rapidly accumulating knowledge about the multiple facets of the syndrome and firmly established that the vector was a mosquito favored by improper or insufficient public sanitation. Most mothers were poor, declared themselves to be brown or black, were unable to continue working in face of the time needed to care for their children (26), had access to free [strings attached^14^] transportation, and a welfare stipend (BCP, also with strings attached – for those with per capita family income below one-fourth of the minimum wage – and prohibited to have other income sources under penalty of losing the stipend). In short, extra demands coming from needs for care brought on by the presence of a child with Zika Congenital Syndrome (or other conditions) were not enough for the state to recognize rights to a stipend. What is essential to be included is the proof of poverty of the beneficiary, which is carefully overseen with periodic checking by the State. These are administrative practices that assure the insecurity of daily life, imposing limitations on the beneficiary population.

The unfairness of these welfare practices, together with the great visibility, because of the national response to the epidemic situation, led to a national mobilization of associations, together with professionals, researchers, and other allied groups pressuring for special treatment. That resulted in the proposal of a Provisional Measure issued by the President of Brazil in September of 2019 and cosigned by the minister of the economy and the minister of citizenship. That measure, with changes, became law six months later by way of congressional approval. It declared a lifelong monthly one minimum wage stipend (no more regular surveillance) for proven carriers of the Zika Congenital Syndrome (verified by a committee and with use of medical documentation that not all women could put together because it often was retained by the services and researchers). Giving with one hand and taking away with the other, the text defined eligibility for the stipend restricted to those born between 2015 and December 31, 2018, with permission to have other income sources (however, prohibiting any other cumulative compensations related to Zika, and, limiting concession of the stipend for those who had qualified for the established BCP limit.). Diniz calls these conditions “pranks^15^” and other authors (39, 40) corroborate this situation and accompany the partially successful efforts in hearings about the Provisional measure to do away with unjustifiable limitations^16^ when it became legislation approved by Congress. Most limitations were retained. Mothers, association presidents, and aligned researchers, including our research team, testified in favor of the ZCS families in these hearings.

Few mothers of other handicapped children have lifelong stipends, however restricted the stipend may be, so this group of Zika mothers, boosted by the international and national public visibility of the epidemic situation, were able to, at least, reach slightly less unequal conditions than other non-Zika mothers in similar situations. The multiple alignments and the exceptionality of the health and welfare sector of the nation having to respond to the new disease permitted this situation.

### Making Films

As anthropological researchers, our entire team became friends of many mothers as we followed their daily itineraries, from homes and neighborhoods to health appointments and exams, to association events of sociality and distribution of donations, and instructional and informative meetings of health officials and medical specialists instructing parents and testing the health and development of children; in busses, vans and cars back and forth from where they needed to go, carrying bags, holding children, and, in general, helping to solve all sorts of problems. Since we believed that these mothers knew what they needed and wanted, we followed their lead. When these women decided to get their grievances off their chest and accuse mistreatment, we were seldom included in the oft-repeated and condemned category of researchers and journalists who only talked with them when they wanted immediate information, and when it was gotten, would forget the women and children.

It did not seem to us that just following these itineraries, writing about them, and doing interviews would be sufficient participation in our declared objective of “associating a deep knowledge of the experience of women and their family networks affected by the virus to an effort to widen sensitivity and response of the health system in a way in a way in which it incorporates mutual knowledge and collaborative practices.” Since it was evident that high visibility in the special epidemic situation was a positive factor in their being able to make their demands be known and treated, we decided to work with them in an activity that would be entertaining, would qualify them, and also would help to disseminate information about them from their point of view, and not from the point of view of others. We were able to interest ten of these very busy mothers in doing a course on audiovisual productions (with cameras and with cell phones). After some women missed many sessions because of other daily demands, or still felt limited in knowing how to produce their films, and others abandoned the course, we were able to produce five short films with each one dealing with a different subject that was identified mutually and divided between mothers in group discussions and divisions of emphasis (work, dedication of fathers, support groups, transportation, and medication). These were women talking about their experience and demands in dealing with Zika in an attractive and legitimating technical format. The emphasized themes were defined by them as relevant for understanding their condition, the collaboration with technicians in framing, producing images, thinking scripts, and making decisions about final editing and collaborating in the dissemination and discussion of the products. Since it was also registered as a university extension course, the mothers earned certificates for their dedication to studying and producing. New information and new skills circulated both within their communication networks and in the communication networks of others.

These films, with legends, are available at https://www.youtube.co/watch?v=6o6UyOx8tqs and were shown to the general public on a première night, with a session of debates with the five producers forming a panel to answer questions about their films and their situation, and then taken to the specialized and innovative State health secretarial Nucleus for the Support of Children and Families with Microcephaly (NACFM) and shared with 12 sub-regional specialized supporter women (“*apoiadoras*”) on the specifically contracted staff. These women offered an institutional response to the need to follow the Zika syndrome families, forming personalized links with the families and dealing with their daily problems. After this, we also took the films to intersectional heads higher up in the Health and related secretariats, to the national planning agencies, and academic meetings and seminars, and to American and English university seminars. The films were well received by all, with many comments about the very humanitarian, “on the ground” viewpoint, and on the eloquence of the mothers. Our original idealization of creating one or more live situations in which public authorities and professionals could become spectators in a full auditorium in which the mothers and families were the speakers certainly went against the grain of time use and hierarchy retention practice of these authorities, but the technical authority of the film, and the firm support of our research group was able to increase the sympathy of authorities in hearing what the women had to say. We certainly believe that in all our activities, we were able to make up part of the multiple alignments which contributed to a diminished inequality for these women.

In short, the films have several advantages: deepening research information, strengthening the skills and self-esteem of mothers, valuing the experience and understanding of mothers, and serving as tools for sharpening consciousness of needs for services. They were not on our original research agenda. It is an example of how the mutual alignment of the research team and the overburdened caregiving mothers led to the creation of additional activity for some women to include in their therapeutic itineraries understandable as contributing to “power with” practices that may assuage gender inequality.

## Final Considerations: What Works and Why?

It is very important to differentiate between gender difference and gender equality. Combating gender inequality does not annul gender differences. What needs to be done in order to confront threatening power domains is what Richard Adams long ago (41) called “symbolic inversion,” and which feminists have taken as an important tactic of valuing the demands of women. What is dealt with as drudgery or is used to deride downgraded groups is exactly what may be used to mark a positive difference. For overburdened mothers who must care for severely handicapped children, motherhood is associated with elevated emotions of dedication Zika Syndrome children however overwhelming that may seem. In general, these are not associated with an abstract list of ways to become “equal.” What works is to value what women are doing and to form strong and multiple alignments with their points of view in a way in which the inequalities they face may be partially allayed without degrading what they are doing.

It is important to recognize that the research objectives of enhanced dialogue between mothers and families and service providers are different objectives of diminishing gender inequality, even if they offer rich intersections. Despite its magnitude, the research seems to have dealt with too few people, too many themes and has left many things to be done. Too few people, because it only dealt with one state in a nation of affected families, because those families most unreachable by associations and service providers in more recondite locations are under-represented, and because there are many caregivers who are not mothers but are important for Zika Syndrome children and have special situations related to gender and generation (fathers, stepfathers, brothers, grandmothers, sisters). Too many themes because, as much as we collaborated in creating a cohesive and firmly objective-centered team, each of our researchers was faithful to their own choice of a path of discovery that required new literature, concepts, and discussions that it would be unfeasible to deal with in this study but are, indeed, relevant for the subject. Obviously, the same can be said for things undone, but what may be especially rewarding would be (1) long-term research that deals with new developmental and social contexts more and more distant from the epidemic origins, (2) a comparison with other epidemic situations emphasizing caregiving and enhanced valuing and burdening of motherhood, (3) a more extended discussion with diverse feminist perspectives, and (4) an ample discussion of caregiving and engaged research.

Doing research as anthropologists interested in the practice of care is no different than taking the ontological (not just methodological) standpoint that becoming involved in daily activities of those who are being researched makes it more unlikely that research practice will be seen by others as an imposition. The discovery of multiple alignments (in which the alignment with anthropologists is just one more, but also reinforces the others) shows how women construct their strategies of attainment of what may make their life experience more tolerable (not to say, better). Mentioning the diverse contexts in which mothers from multiple alignments: counterposing seminar discussions on the value of abortion without losing sight of the importance of care as a feminist demand in their situation, using formal priorities of an epidemic situation and favors of sympathetic professionals to get more immediate and complete treatment, selectively choosing a limited number of mostly-female relatives and friends for daily support, participating in associations dedicated to their “bio-identity” as mothers of children with special needs, including themselves in judicializing demands to get more enduring and favorable conditions, and producing films from the points of view of their experience to be seen and understood by others. Each agent in each bundle of alignments brings the use of their expertise and social networks to play in how Zika Syndrome mothers may have their situation improved, and in turn, these women collectively will gather around those capable of turning these connections and knowledge into something they judge to be capable to contribute to actions which may be characterized as working in a “power with” direction.

Such alignments are not done indiscriminately. They are lived with an eye to the avoidance of the prevalent practice of administered insecurity in which the hierarchization of agents and their domains of activities creeps back into the relation, threatening to reinforce inequality. They recreate barriers and limitations and make the experience of searching for greater gender equality an arduous task of constant reinstatement of the importance of valuing the specific differences and contexts in which women find themselves. This also requires vigilant attention to the removal or diminishing of what conveys continued insecurity and degradation for them.

Zika Syndrome mothers are overburdened and will continue to be, however, favored by the visibility of priorities given to an epidemic situation. Their insertion into a multiplicity of alignments makes it possible to say they have become involved in a process of reducing gender inequality carrying their demands to public spaces and institutional settings. Some agents in these sectors have become more aware of their plight as caregivers and citizens. Returning to our first question, for the women described here, it is possible to say that even though affected by an event that heightens gender inequality, they also may be seen as having been reinforced. It is important to remember that the description of these mothers showed that for several reasons, not all Zika mothers get involved in the multiple alignments and those who do have varying degrees of involvement. But in the case of Pernambuco, with strong associations and many therapeutic alternatives, most of the mothers can be seen as having found new ways of building power with other women in numerous situations that require them to act as mothers. This is unquestionable for dozens of women with whom we interacted.

If the film-making promoted by the research team directly involved a reduced number of these women, their diverse activities and films reached out to a much larger contingent and pointed out ways that turn their experience into something positive. However, the positive production of films by these mothers was for promoting dialogue between mothers and service administrators. It is the confidence of a multi-fronted alliance as reliable friends that worked for our contribution to diminishing gender inequality for these women, as varied as they may be. Much more important than the films themselves, and what is conveyed in the films because of what was learned by the co-experience of researchers and Zika mothers, was the discovery of the skills of these women to create multiple alignments in the redefinition of their life courses that the Zika epidemic required. This heavily unequal context of an epidemic that starts in the womb and throws principally young mothers into a world of seemingly unending health treatment practices is an important event for understanding the multiple configurations of gender and power. There are very tangible gains to be found in a mutual alignment of women among themselves and with important institutional and relational reinforcements that make it possible to affirm, as well, that this event can serve to construct political awareness and practice associated with demands for services, be they from the state or other agents. The greatest challenge is to keep the exercise of informed citizenship alive as time changes priorities and continue a sharp awareness of, and resistance to, state and other institutional agents who are prone to administer insecurity.

## Data Availability Statement

The datasets presented in this article are not readily available due to identifiable written notes and transcriptions. Requests to access the datasets should be directed to rparryscott@gmail.com.

## Ethics Statement

The studies involving human participants were reviewed and approved by Ethics Commitee of the Federal University of Pernambuco - Brazilian National Platform - CAAE: 65741717.0.0000.5208 Secretaria Estadual de Saúde - Pernambuco - 02/08/2017. The patients/participants provided their written informed consent to participate in this study. Written informed consent was obtained from the individual(s) for the publication of any potentially identifiable images or data included in this article.

## Author Contributions

PS was the principal author and accountable for the content of this work.

## Funding

The research Project, Etnografando Cuidados e Pensando Políticas de Saúde e Gestão de Serviços para Mu- lheres e Seus Filhos com Distúrbios Neurológicos Relacionados com Zika em Pernambuco, Brasil was coordinated by PS of FAGES (Núcleo de Família, Gênero, Sexualidade e Saúde-Study Group of Family, Gender, Sexuality and Health) of the Universidade Federal de Pernambuco, financed by CAPES (8888.130742/2016-01), CNPq (440411/2016-5), and DECIT-MS. Additionally, with the title of Action Ethnography on Care, Disability and Health Policy and Administration of Public Service for Women and Caretakers of Zika vírus affected Children in Pernambuco, Brazil, financed by FACEPE/Newton Fund (APQ 0553-7.03/16). Funding was for research team stipends and field expenses and participation in meetings. Brazilian Open Access policy discourages payment fees for individual authors as a general rule in its evaluation system, therefore, they ask for such fees to not be included in grant requests. Brazian salaries are low and getting lower with the exchange rate. I hope to receive a waiver of fees.

## Conflict of Interest

The author declares that the research was conducted in the absence of any commercial or financial relationships that could be construed as a potential conflict of interest.

## Publisher's Note

All claims expressed in this article are solely those of the authors and do not necessarily represent those of their affiliated organizations, or those of the publisher, the editors and the reviewers. Any product that may be evaluated in this article, or claim that may be made by its manufacturer, is not guaranteed or endorsed by the publisher.

## Footnotes

^1^The WHO fact sheets present updated information on the Zika Virus (https://www.who.int/news-room/fact-sheets/detail/zika-virus (access May 20, 2021).

^2^The downplaying of the importance of sexual transmission in public prevention campaigns and the lack of attention to the importance of both female control of negotiations about sexual health and fertility regulation, and the lesser attention to these questions given by men, are shown to contribute to an intensification of gender inequality in relation to reproductive health, concentrated among the more vulnerable lower-income populations in structurally and environmentally unhealthy areas by Coutinho et al. (3).

^3^This argument is developed more extensively in a dossiê organized by the author of this article (8).

^4^These may best be understood, in the terms of Vainer and Araujo (14), as large Investment projects, clearly less concerned about the impacts on the local population than on the prospect of providing products to geographically and socially distant populations in a way which brings positive returns to the investors.

^5^Evidently here, references are to ideas on reciprocity and redistribution of Mauss (15) Polanyi as re-edited in and Dalton (16) and Sahlins (17), as well as many authors whose perceptions derived from these authors.

^6^An excellent example of the cumulative effects of administrative neglects and violence on the day-to-day life of a mother with a handicapped child needing multiple special attention, besides highlighting the daily despair of repeated frustrations, delays, and absences, also were elements in creating a courageous and battling mother (21).

^7^The research Project, “Etnografando Cuidados e Pensando Políticas de Saúde e Gestão de Serviços para Mulheres e Seus Filhos com Distúrbios Neurológicos Relacionados com Zika em Pernambuco, Brasil” is coordinated by Russell Parry Scott of Family, Gender and Sexuality Research Group INucleo de Estudos Família, Gênero e Sexualidade (FAGES) (Núcleo de Família, Gênero, Sexualidade e Saúde-Study Group of Family, Gender, Sexuality and Health) of the Universidade Federal de Pernambuco, financed by Coordination of Personnel of Higher Education [Coordenação de Aperfeiçoamento de Pessoal de Educação Superior (CAPES)] (8888.130742/2016-01), Brazilian National Council for Scientific and Technological Development [Conselho Nacional de Pesquisa Científica e Tecnológica (CNPq)] (440411/2016-5), and DECIT-MS. Additionally, with the title of “Action Ethnography on Care, Disability and Health Policy and Administration of Public Service for Women and Caretakers of Zika vírus affected Children in Pernambuco, Brazil,” financed by Pernambuco State Foundation for the Support of Science [Fundação de Amparo á Ciência e Tecnologia de Pernambuco (FACEPE)]/Newton Fund (APQ 0553-7.03/16).

^8^The single authorship of this paper is in common agreement among the many co-producers of information and interpretations of our research team, named above. When we write together, those who wrote are authors and the others are recognized as co-producers. They include Marion Quadros, Ana Claudia Rodrigues, Luciana Lira, Silvana Matos, Fernanda Meira, Raquel Lustosa Alves, Ana Letícia Veras, Diego Oliveira, Thaíza Vasconcelos, Katarina Brito, and Laís Santos e Gabriela Ribeiro. It is uncommon in anthropological research to have large teams of co-authors.

^9^The FIOCRUZ foundation is the largest collective health institution in the country. It is based in Rio de Janeiro and has branches in campuses in 10 Brazilian states and in Maputo, Mozambique (https://portal.fiocruz.br/unidades-e-escritorios accessed on May 20, 2021).

^10^Especially in the beginning of the epidemic, the unknown consequences of the Zika virus were unlikely to pose the question of abortion for many women. Even more so because of the restrictive nature of Brazil's legislation (30, 31), which contributed to such practice being dealt with as illegal abortion is banned in Brazil unless a woman has been raped, her life is in danger, or the fetus has anencephaly, a fatal brain disorder. In any case, when you are together with dozens of women who gave birth to their children with the Zika syndrome, it is felt to be an offensive attack on both the mother and the child to say “You should have aborted”. That occurs together with women remembering some occasions when they refused assistance which was offered to them after the implications of Zika on infants became well-known.

^11^The abbreviated way that many women say this is feeling that they are “cobaias” (guinea-pigs).

^12^Mortality is high, Barbosa et al. (26) reported that up to the end of 2018, 9% of the infants born with the Zika Congenital Syndrome in Pernambuco died.

^13^This is not the place to develop the idea of care domains [see (25, 36) for a discussion of how different versions of understanding what care is are performed in relational, treatment/services and knowledge].

^14^Only for therapy, not for other appointments, without rights for accompanying helpers, cancelled if used improperly or missed scheduled travel, among other restrictive rules (38).

^15^“*Pegadinhas*” is how Diniz twitted a comment on the Provisional Measure to call attention to these limitations.

^16^The text, which had been proclaimed by the president as unchangeable under the threat of rescinding the measure, was, in fact, modestly changed after congressional committee hearings in which our research team participated directly with the testimony of Silvana Matos (28, 32).
